# The protein corona protects against size- and dose-dependent toxicity of amorphous silica nanoparticles

**DOI:** 10.3762/bjnano.5.151

**Published:** 2014-08-27

**Authors:** Dominic Docter, Christoph Bantz, Dana Westmeier, Hajo J Galla, Qiangbin Wang, James C Kirkpatrick, Peter Nielsen, Michael Maskos, Roland H Stauber

**Affiliations:** 1 Molecular and Cellular Oncology, ENT/University Medical Center Mainz, Langenbeckstr. 1, 55101 Mainz, Germany; 2 Fraunhofer ICT-IMM, Carl-Zeiss-Str. 18-20, 55129 Mainz, Germany; 3 Institute of Biochemistry, Westfälische Wilhelms-University, Wilhelm Klemm-Str. 2, 48149 Münster, Germany; 4 Suzhou Institute of Nano-Tech and Nano-Bionics, Chinese Academy of Sciences, Suzhou, 215123 China; 5 Institute of Pathology, University Medical Centre, Institute of Pathology, Langenbeckstr. 1, 55101 Mainz, Germany; 6 Department of Biochemistry and Molecular Cell Biology, University Medical Center Hamburg-Eppendorf, Germany

**Keywords:** biobarrier, gastrointestinal tract, high-throughput profiling, nanomedicine, nanotoxicity

## Abstract

Besides the lung and skin, the gastrointestinal (GI) tract is one of the main targets for accidental exposure or biomedical applications of nanoparticles (NP). Biological responses to NP, including nanotoxicology, are caused by the interaction of the NP with cellular membranes and/or cellular entry. Here, the physico-chemical characteristics of NP are widely discussed as critical determinants, albeit the exact mechanisms remain to be resolved. Moreover, proteins associate with NP in physiological fluids, forming the protein corona potentially transforming the biological identity of the particle and thus, adding an additional level of complexity for the bio–nano responses.

Here, we employed amorphous silica nanoparticles (ASP) and epithelial GI tract Caco-2 cells as a model to study the biological impact of particle size as well as of the protein corona. Caco-2 or mucus-producing HT-29 cells were exposed to thoroughly characterized, negatively charged ASP of different size in the absence or presence of proteins. Comprehensive experimental approaches, such as quantifying cellular metabolic activity, microscopic observation of cell morphology, and high-throughput cell analysis revealed a dose- and time-dependent toxicity primarily upon exposure with ASP30 (Ø = 30 nm). Albeit smaller (ASP20, Ø = 20 nm) or larger particles (ASP100; Ø = 100 nm) showed a similar zeta potential, they both displayed only low toxicity. Importantly, the adverse effects triggered by ASP30/ASP30L were significantly ameliorated upon formation of the protein corona, which we found was efficiently established on all ASP studied. As a potential explanation, corona formation reduced ASP30 cellular uptake, which was however not significantly affected by ASP surface charge in our model. Collectively, our study uncovers an impact of ASP size as well as of the protein corona on cellular toxicity, which might be relevant for processes at the nano–bio interface in general.

## Introduction

Besides the wide use of nanomaterials in industrial products, biomedical applications of nanoparticles (NP) are steadily increasing [1–5]. However, despite intense investigations, current knowledge of the physiological effects of nanoparticles on biological barriers and the underlying molecular mechanisms is still fragmented [6–8]. The main purpose in the field of nanotoxicology is to address potential adverse health effects induced by such novel nanomaterials [8]. Owing to their high surface free energy and their high surface area-to-volume ratio nanoparticles are highly reactive. Such a high reactivity to various biotic and abiotic environments, particularly the interaction of nanomaterials with biological systems and their unique physico-chemical properties may potentially result in yet unknown toxic effects [8].

The respiratory system and the gastrointestinal (GI) tract are considered to be the main routes by which NP may access the body [6–8]. Nanomaterials reach the GI tract mostly by ingestion of NP-containing products or upon direct biomedical application as contrast agents or drug delivery devices [9–10]. From our experience in pharmaceutical and medical history, we have learned that oral delivery is the preferred administration route for patients [9–10]. Similar to the lung, also the GI tract is a major biobarrier target organ for nanoparticles due to its huge surface area. Also, not only the lung but also the GI tract is performing multiple functions, is specialized in the uptake and excretion of various molecules and thus, connects the environment to the bloodstream. A well accepted in vitro model to study NP exposure via the oral route is the epithelial colonic carcinoma cell line Caco-2, which has features consistent with differentiated small intestinal enterocytes [11–12].

Silica-based NP are not only widely used in food products [13–14], but have also attracted much attention for biomedical applications as imaging moieties and drug carriers [10,15]. Amorphous silica is registered as a food additive within the EU, named also E551, and therefore it is already widely used in various consumer products [9–10,15]. The assessment of amorphous silica being non-toxic is mostly based on the testing of micrometer-sized bulk materials [16]. Whether nano-sized amorphous silica, and such ultra-small materials in general, should be considered as a completely novel entity of materials is still an ongoing debate in the growing field of nanotechnology [14,17–18]. Silica NP offer great potential for various applications due to their unique properties such as the variety of surface modifications and their convenient synthesis [9–10,15]. Though, the biological influence of such type of NP and its correlation with the physico-chemical properties of the nanomaterial, such as size, density, and surface chemistry are still not understood in detail [19–21]. In addition, due to their high surface free energy, nanomaterials, including silica-based NP, adsorb (bio)molecules upon contact with biological or abiotic environments, forming the so-called corona [22–23]. Particularly, the biophysical properties of particles covered by a protein corona may differ significantly from those of the formulated particles and thus, seem to critically define the biological identity of the particles [24]. Thus, numerous studies have been conducted to generally dissect and mechanistically understand the formation and kinetic evolution of the protein corona, its dependence on the physico-chemical properties of the nanoparticles as well as its (patho)biological relevance [7,22,25–29]. Albeit the protein corona has been shown to impact (patho)biological processes at the nano–bio interface, the molecular mechanisms are still not yet resolved [22]. Consequently, the presented study investigated the potential toxic effects of different amorphous silica NP (ASP), focusing on particle size and the relevance of the protein corona.

## Results

### Characterization of amorphous silica nanoparticles (ASP)

**Figure 1:**
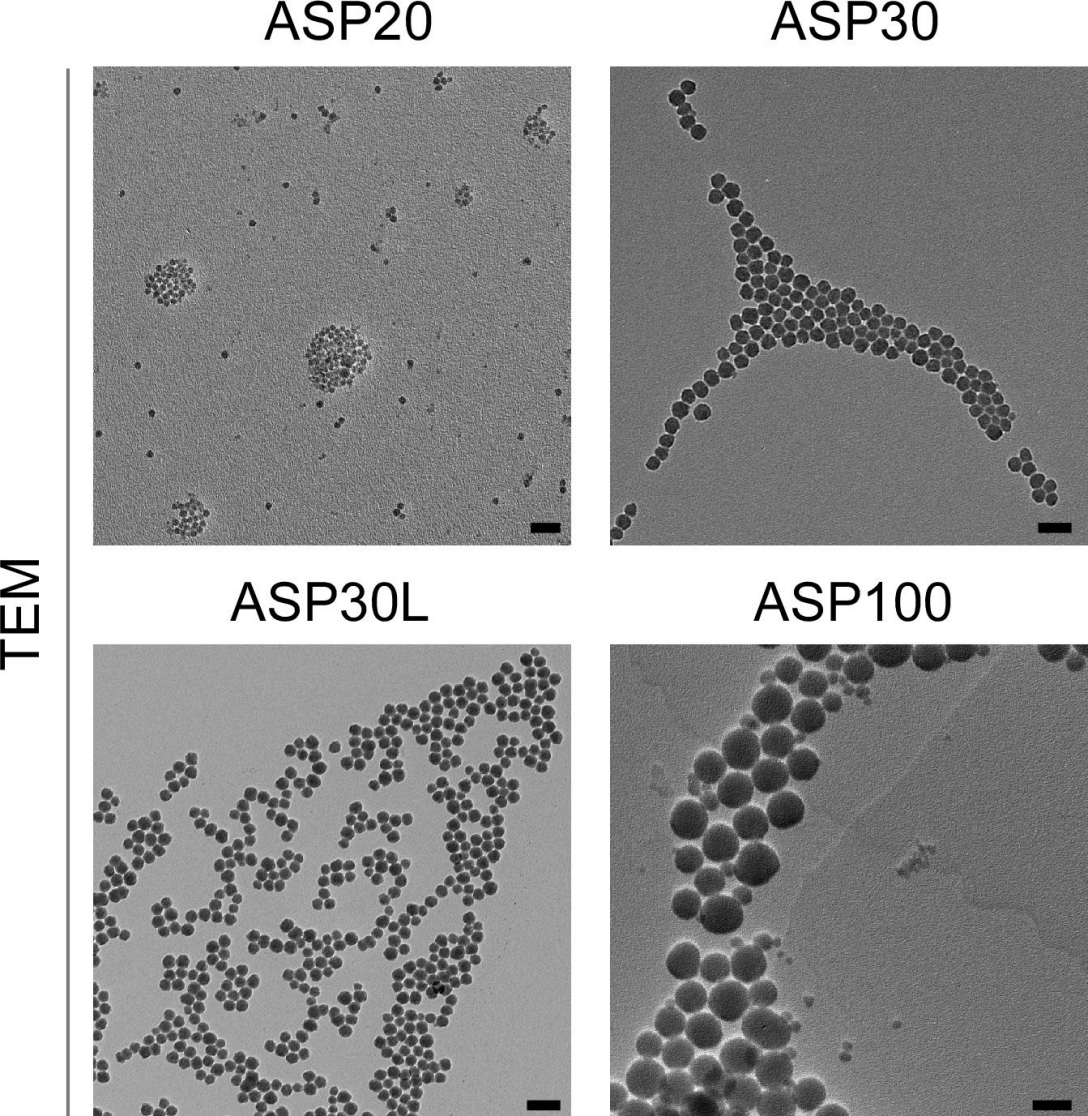
Transmission electron microscopy (TEM) images of representative ASP used in the study. Scale bar = 100 nm.

A comprehensive characterization of physico-chemical characteristics of nanomaterials is an absolute prerequisite for the subsequent experimentation. Hence, we analyzed critical properties of the ASP, such as their size distribution and surface charge, in the presence and absence of serum proteins by independent experimental methods. First, the size, spherical shape and homogeneity of the ASP were visualized by transmission electron microscopy (TEM) (Figure 1). Next, we examined the stability of the ASP dispersions in water, salt-containing buffer (buffer A), and cell culture medium (DMEM) with or without the addition of 10% fetal calf serum (FCS). According to dynamic light scattering (DLS) and zeta potential measurements, all ASP display the expected hydrodynamic diameter and carry negative surface charges in water, as reﬂected by their negative zeta potential (Table 1). As the highest absolute values for zeta potential were measured in water, the colloidal stability is expected to be best when the surface-charge-stabilized silica particles are dispersed in water containing low concentrations of salt. Nevertheless, all presented particles show hydrodynamic diameters in the range of the primary particle size also in buffer A and in DMEM without FCS. Exposure to serum proteins however resulted in a significant increase in the average hydrodynamic diameter of the particles and in a decrease in the absolute value of their zeta potential, most likely due to the formation of the protein corona as well as due to aggregation.

**Table 1:** Physico-chemical characterization of ASP used in the study. The average size of the different ASP was determined in dry state (TEM) as well as in water, buffer A, and DMEM with or without 10% FCS by angular-dependent DLS measurements with a goniometer setup by ALV. The values of µ_2_ as a measure for the size polydispersity are derived directly from the cumulant analysis of DLS autocorrelation data. As a rough estimation, µ_2_ values smaller than 0.05 indicate a strict monodisperse size distribution, whereas µ_2_ values above 0.1 are indication for broad size distributions. Zeta potentials were determined with a Zetasizer system. Values are mean ± SD from three independent experiments.

particle	TEM diameter ± SD [nm]	DLS hydrodynamic diameter<*D*_h_>_z_ [nm] / µ_2_				Zetasizer system zeta potential *z* [mV]			

	in dry state	water	buffer A	DMEM	DMEM/10% FCS	water	buffer A	DMEM	DMEM/10% FCS

**ASP20**	19.2 ± 4.4	31.0 / 0.12	25.0 / 0.19	24.2 / 0.15	122.6 / 0.2	−58	−11	−26	−12
**ASP30**	31.4 ± 3.8	32.8 / 0.12	36.2 / 0.12	35.2 / 0.10	141.6 / 0.11	−53	−17	−20	−12
**ASP30L**	31.2 ± 4.0	32.4 / 0.06	34.6 / 0.04	32.8 / 0.07	198.6 / 0.16	−57	−20	−27	−12
**ASP30F**	30.6 ± 6.8	33.4 / 0.06	33.6 / 0.05	33.6 / 0.06	138.7 / 0.12	−56	−15	−21	−9
**ASP30F-COOH**	27.2 ± 3.8	28.0 / 0.09	29.0 / 0.08	28.2 / 0.07	132.8 / 0.11	−58	−18	−25	−14
**ASP100**	109.8 ± 34.4	144.0 / 0.03	141.2 / 0.05	142.6 / 0.05	172.8 / 0.10	−55	−26	−32	−11

### ASP affect cell vitality in a size- and dose-dependent manner

**Figure 2:**
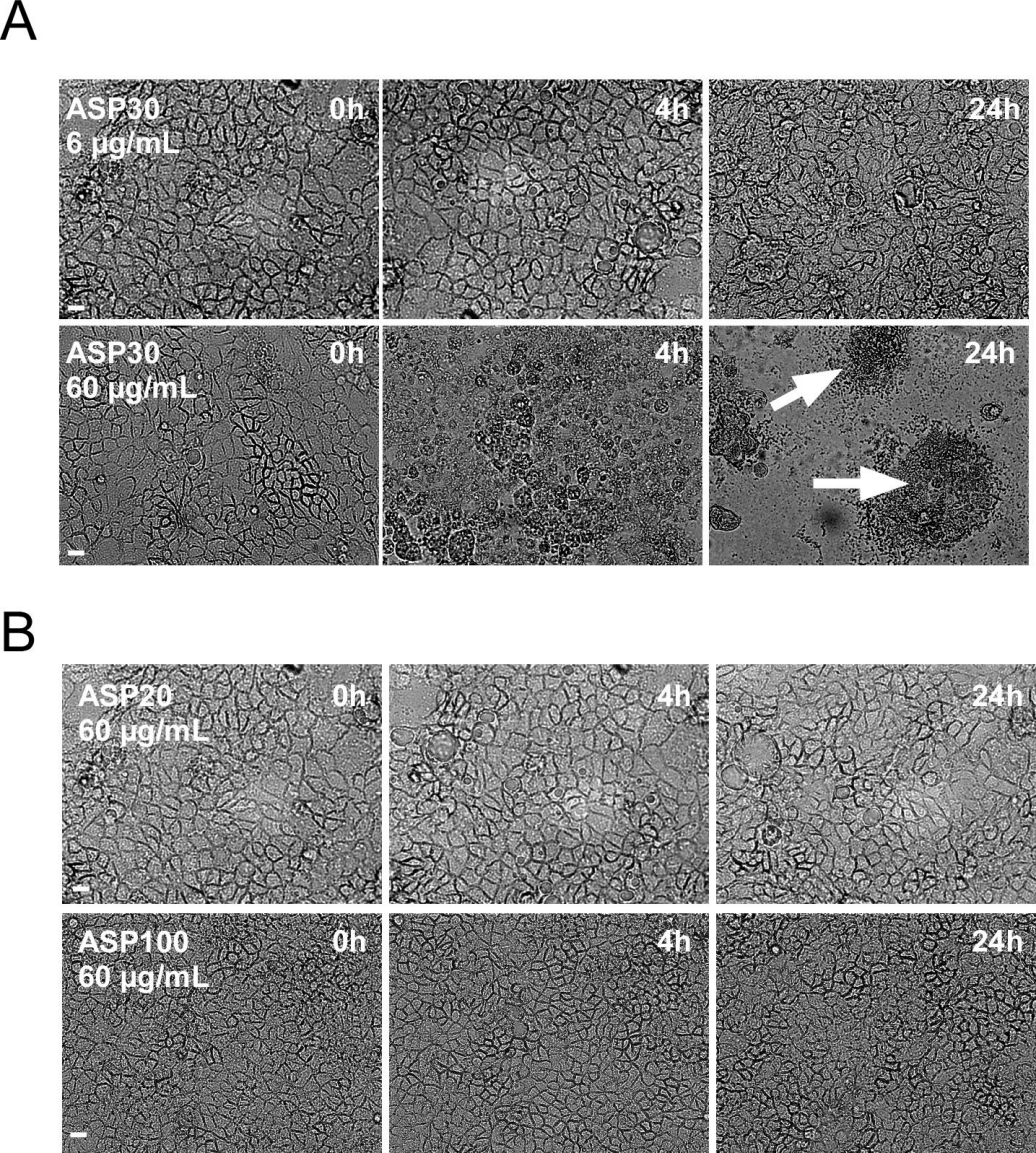
Microscopy-based assessment of cell vitality by analyzing ASP-induced morphological changes. (A/B) Caco-2 morphology was recorded by phase contrast microscopy. Cells were exposed to the indicated doses of the different ASP ((A) ASP30 and (B) ASP20 or ASP100) for 4 h in serum-free DMEM. Subsequently, cells were washed twice with DMEM and further cultivated in DMEM containing 10% FCS for 20 h prior to microscopic inspection. Scale bar = 10 µm. Arrows highlight loss of adhesion and cell clumping as signs for toxicity.

To investigate the (patho)biological effect of the ASP, we used independent experimental approaches. As a rapid and inexpensive screening method for cytotoxicity, we first employed light microscopy to analyze morphological changes of the Caco-2 cells following exposure to the different ASP (Figure 2). Exposure to ASP30 or ASP30L under serum free conditions induced dose- and time-dependent significant morphological changes, such as loss of a structured cell shape, disruption of the monolayer, and loss of adhesion, which is indicative of an impaired cell vitality (Figure 2A and not shown). The ASP30 impact on cell vitality was most prominent 24 h after exposure to ASP30 (Figure 2A, marked by arrows). Interestingly, such effects were not observed when cells were exposed even to high doses (60 µg/mL) of either the smaller (ASP20, Ø = 20 nm) or larger particles (ASP100; Ø = 100 nm) under identical experimental conditions (Figure 2B).

**Figure 3:**
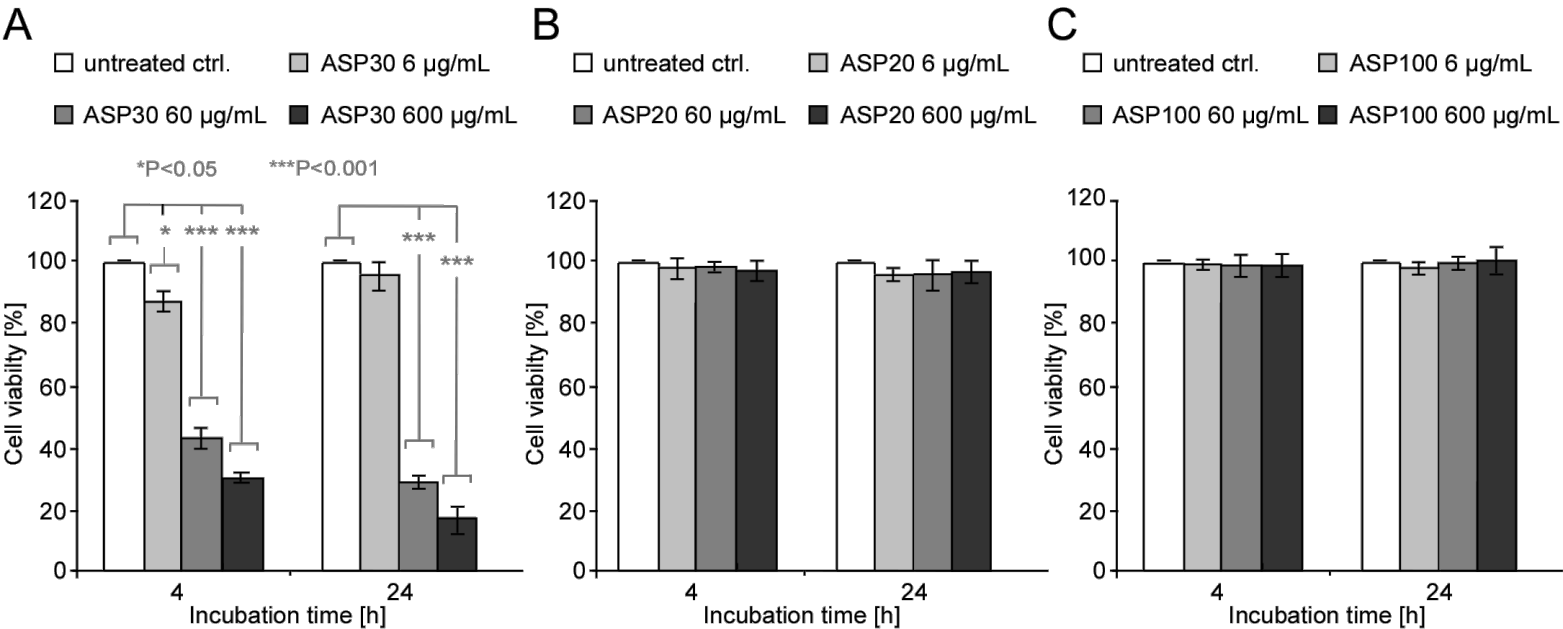
Impact of ASP30 on the cellular metabolic activity. (A/B/C) Caco-2 cells were incubated with the indicated concentrations of ASP30 (A), ASP20 (B) and ASP100 (C) in serum-free DMEM and analyzed after 4 or 24 h by using the MTT assay. Data are depicted as percentage compared to untreated control cells, which was set to 100% vitality. Results are shown as means ± SD (*n* = 3). *P < 0.05, **P < 0.01 and ***P < 0.001.

These results could be confirmed by independently assessing the metabolic activity of the cells using the MTT biochemical assay (Figure 3). This colorimetric assay measures the activity of cellular enzymes that reduce the tetrazolium dye MTT to its insoluble formazan in living cells. Compared to untreated control cells, exposure to ASP30 was found to significantly reduce the cell vitality in a dose- and time-dependent manner (Figure 3A), whereas no effects were observed upon treatment with ASP20 (Figure 3B) or ASP100 (Figure 3C). These results not only confirm the size- and dose-dependent ASP toxicity but also underline the reliability of the microscopy-based morphological assay as a convenient approach to test for nanotoxicity.

**Figure 4:**
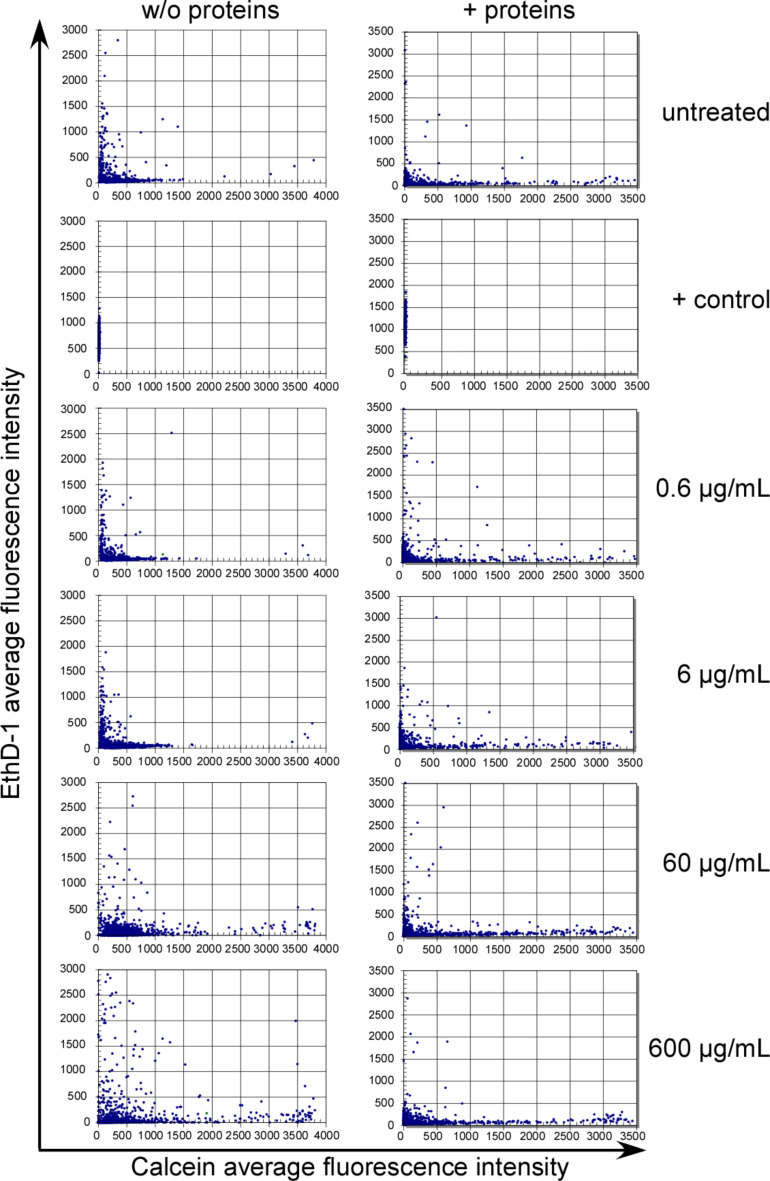
Automated microscopy to analyze the impact of exposure to ASP30 on the cell viability. Caco-2 cells were exposed in DMEM to the indicated ASP30 concentrations for 4 h in the absence (w/o protein; left panel) or presence (+-proteins; right panel) of 10% FCS, washed with buffer A, and the vitality was evaluated by using the two-color fluorescence assay. Intensity of calcein (living cells; green) and ethidium homodimer-1 (dead cells; red) signal was monitored using the Cellomics ArrayScan^®^ VTI. A minimum of 500 cells were analyzed per well, and each treatment was done in triplicate. As a positive control, cells were treated with methanol (+ control), resulting in 100 % damaged cells.

**Figure 5:**
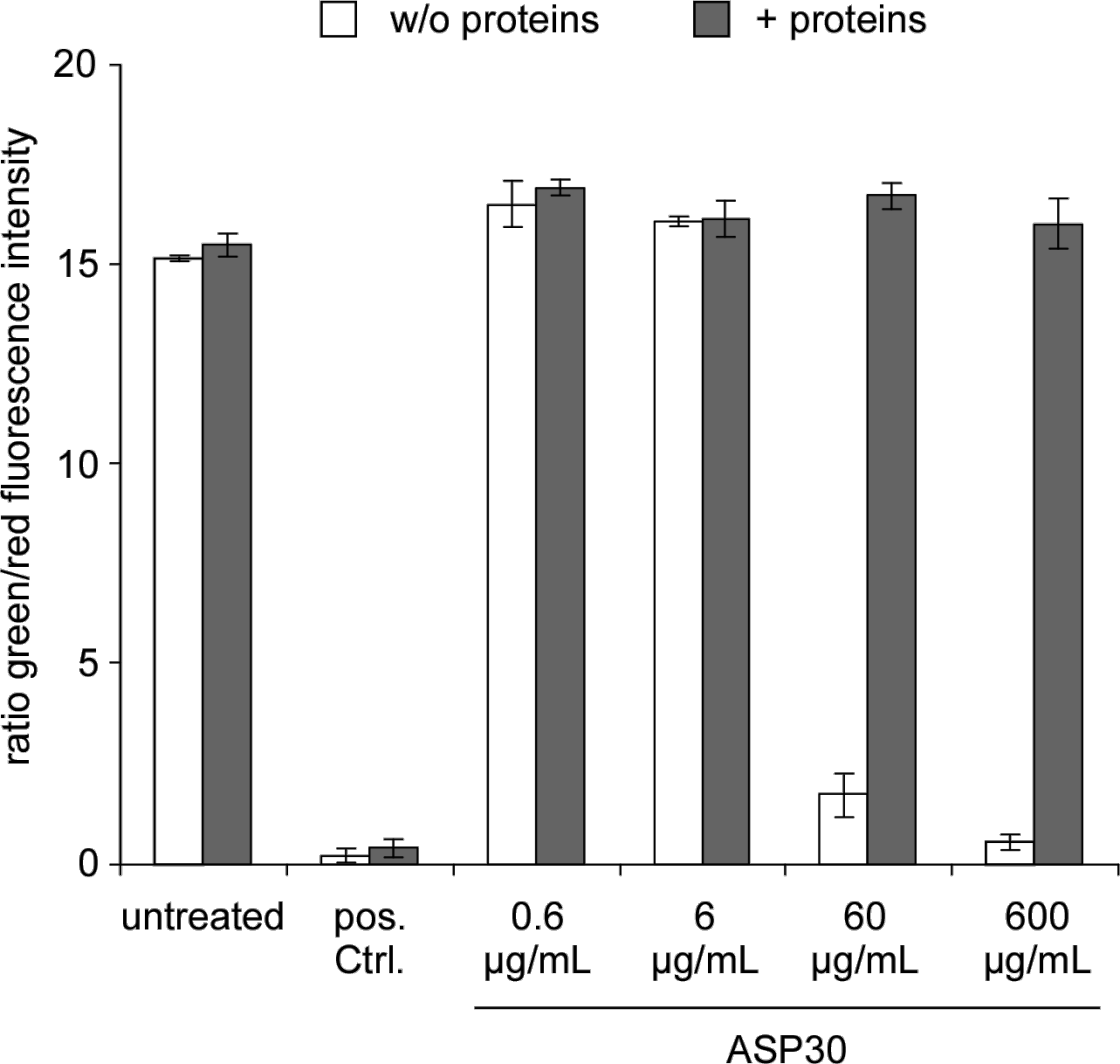
Quantification of the impact of ASP30 on the cell viability. Determination of the ratio of the average calcein (living cells; green) to ethidium homodimer-1 (dead cells; red) signals according to Figure 4. As described in Figure 4, CaCo-2 cells were exposed to the nanoparticles suspended in DMEM (open rectangles) or DMEM containing 10% human plasma (filled rectangles) for 4 h and analyzed by automated microscopy. Columns, mean; bars, ± SD from three independent experiments.

Currently, high-throughput testing is actively discussed as a key strategy to systematically establish nanomaterial structure–activity relationships (nanoSAR) [30]. Such assays and respective platforms are required to investigate the sheer endless number of bio-physico-chemical interactions occurring at the nano–bio interface. Besides already applied enzymatic/biochemical assays [30], we, here, present an automated high-throughput microscopy based approach, generally applicable to reliably and reproducibly assessing the cell vitality following exposure to nanomaterial. By uUsing the ArrayScan^®^ VTI fluorescence microscopy imaging platform [31], we established a dual-color fluorescence cell vitality assay. By employing fluorescent probes that recognize cell viability by measuring intracellular esterase activity (calcein-AM; green) as well as plasma membrane integrity (ethidium homodimer-1/EthD-1; red), the assay allows for the simultaneous quantitation of live and dead cells by fluorescence microscopy. Only living cells are able to convert the virtually non-fluorescent cell-permeable calcein-AM to the intensely fluorescent calcein, resulting in an intense uniform green fluorescence of living cells. EthD-1 is however excluded by the intact plasma membrane of living cells, and only enters cells with damaged membranes. Here, it undergoes a 40 fold enhancement of fluorescence upon binding to nucleic acids, thereby producing a bright red fluorescence characteristic for dead cells. As shown in Figure 4, employing our assay as an additional independent method revealed a dose-dependent loss of cell vitality upon exposure of Caco-2 cells with ASP30. Whereas doses of 0.6 µg/mL or 6 µg/mL ASP30 showed no effect, 60 µg/mL and particularly 600 µg/mL resulted in strong red-fluorescence due to loss of membrane integrity, indicative of dead cells. For assay quantification, we calculated the ratio of the average calcein (living cells; green) versus ethidium homodimer-1 (dead cells; red) intensity signal (Figure 5). Whereas the ratio of living to dead cells remained almost unchanged for the untreated Ctrl. as well as after the treatment with 0.6 µg/mL or 6 µg/mL ASP30 in the presence and absence of proteins, incubation with 60 µg/mL or 600 µg/mL in absence of proteins led to a significant decrease of this ratio, indicative of cell death.

### The protein corona ameliorates ASP-induced toxicity

**Figure 6:**
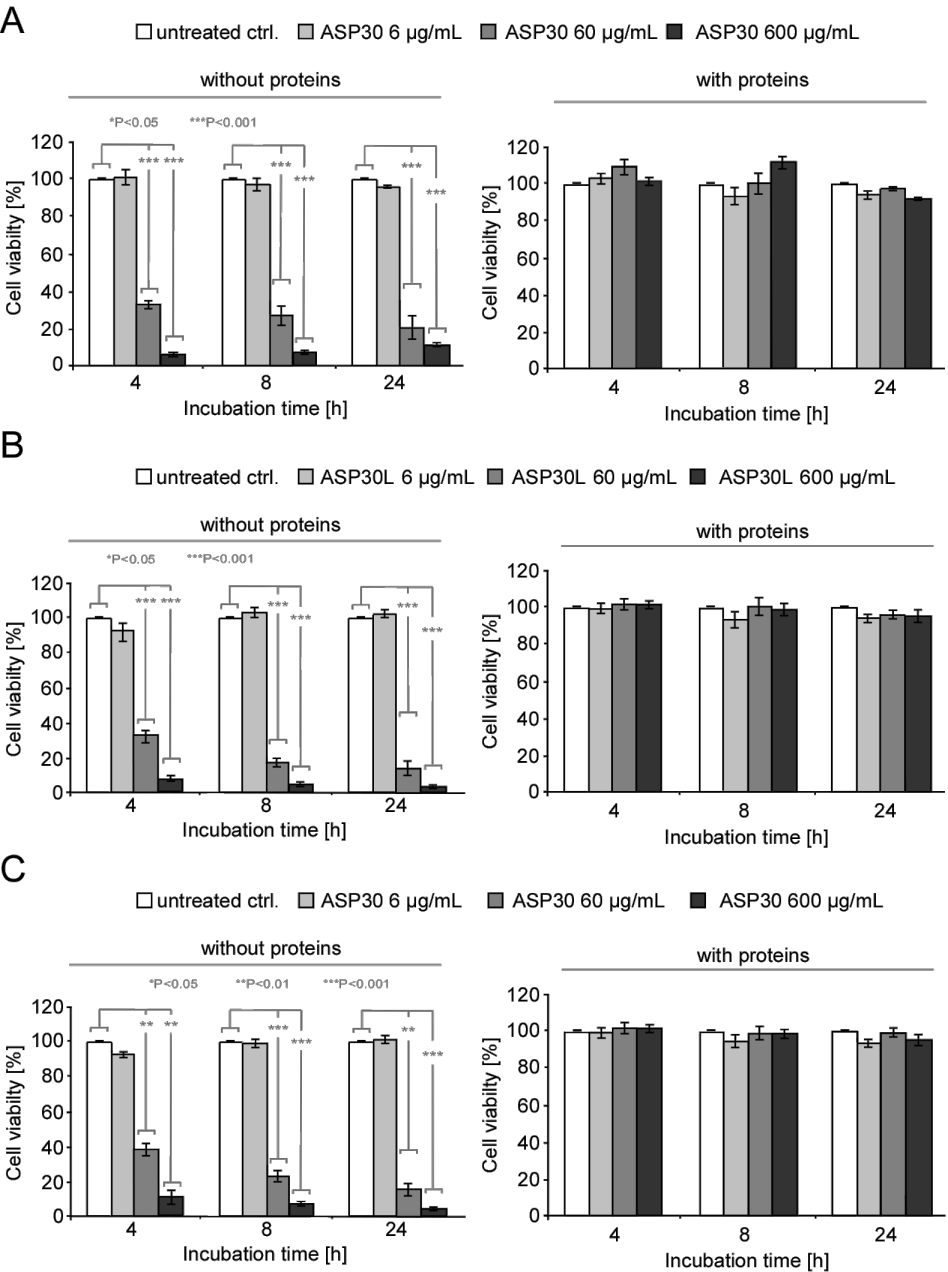
The protein corona ameliorates ASP-induced toxicity in GI-tract models. (A/B) Caco-2 cells or HT-29 cells (C) were treated with different concentrations (6 to 600 µg/mL) of ASP30 (A/C) or ASP30L (B) in serum-free or serum-containing (10% human plasma) DMEM. The cell viability was analyzed for the indicated time points by using the MTT assay. Data are depicted as percentage compared to untreated control cells, which was set to 100% vitality. Results are shown as mean ± SD (*n* = 3). *P < 0.05, **P < 0.01 and ***P < 0.001.

Currently, the protein corona of nanomaterials in general is actively discussed as a major factor (co)determining their biological identity and hence, effects at the nano–bio interface [22]. Thus, we next investigated the impact of the protein corona on the observed toxicity of ASP. Notably, whereas the exposure to ‘pristine’ ASP30 or ASP30L significantly affected cell vitality of Caco-2, automated high-throughput microscopy revealed that cell death was almost completely prevented upon treatment in the presence of serum or human plasma proteins (Figure 4, right panel). Similar results were obtained by assessing the cell viability using independent methods, such as the MTT assay (Figure 6). Similar to the lung surfactant [32], also epithelial cell of the GI tract are covered by an additional biobarrier, i.e., by mucous matrices [33]. To investigate the impact of mucus associated to GI tract cells on the observed effects, we included the mucus-secreting colorectal cell line HT-29 in our study. The HT-29 cell model is a widely accepted model for studying the impact of mucus associated to cells of the GI tract [33]. As shown in Figure 6C, even in the presence of cell-associated mucus the cytoprotective impact of the protein corona was preserved.

### Effect of the protein corona on the cellular uptake

**Figure 7:**
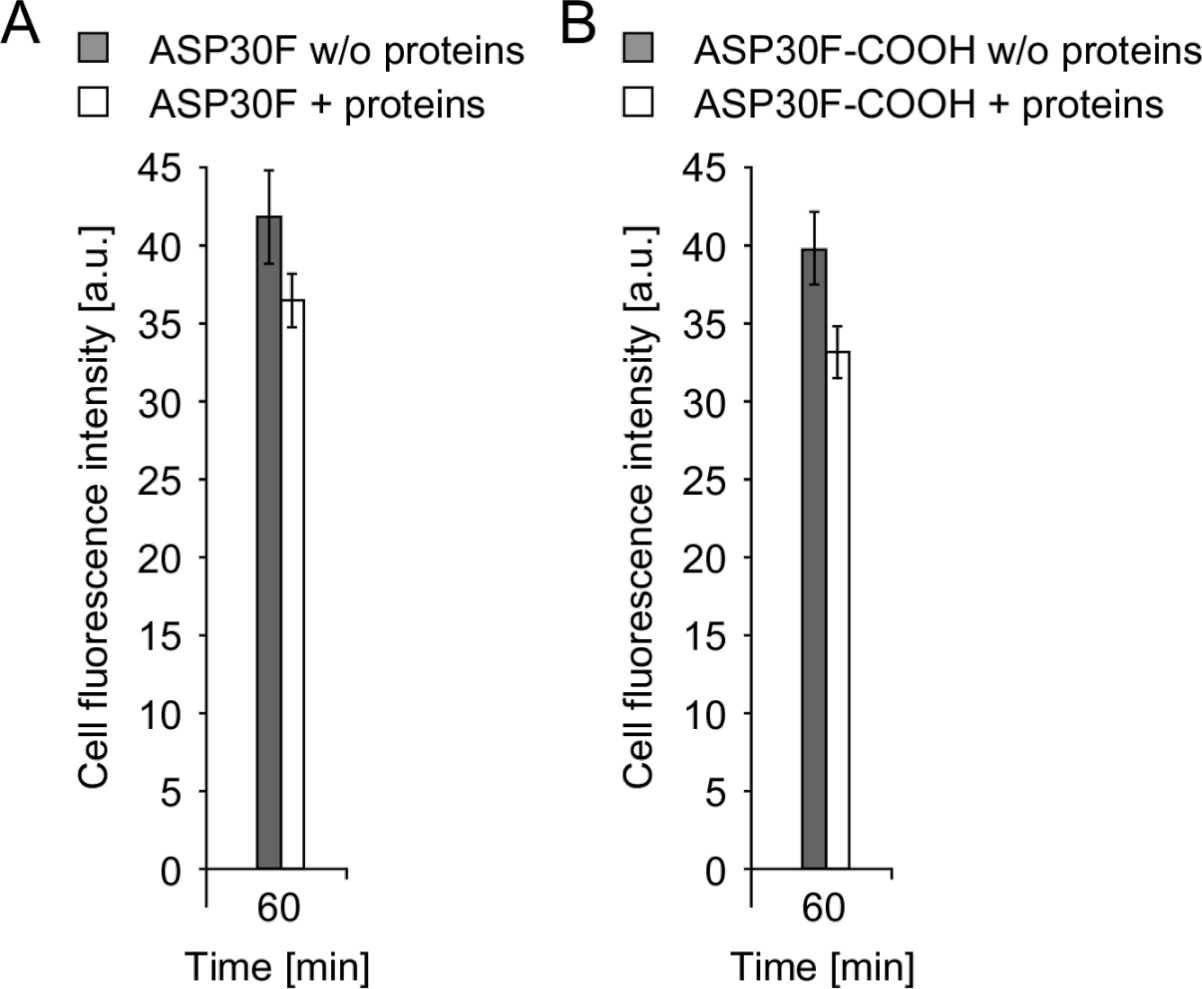
Uptake of fluorescently labeled ASPs. (A/B) Automated microscopy to quantify time-dependent cellular uptake of fluorescent amorphous silica nanoparticles (ASP30F (A) and ASP30F-COOH (B)). CaCo-2 cells were exposed to the nanoparticles (100 µg/mL) suspended in DMEM (filled rectangles) or DMEM containing 10% human plasma (open rectangles) for 60 min. Total cellular fluorescence is displayed as arbitrary units (AU). Assays were performed in triplicates. Columns, mean; bars, ± SD from three independent experiments.

Nanoparticle uptake is an important determinant for nanopathology [22,34–35]. To investigate the effect of the protein corona on cellular uptake mechanisms we analyzed the uptake of two fluorescently labeled ASPs with comparable physico-chemical characteristics (ASP30F and ASP30F-COOH; Table 1). As shown in Figure 7, automated microscopy revealed that in the absence of proteins slightly more ASPs were attached and taken up by the Caco-2 cells compared to the incubation in the presence of proteins after 60 min (Figure 7A/B). By increasing the surface charge by carboxylation (ASP30F versus ASP30-COOH; Table 1) we did not, however, observe a significant difference in cellular uptake (Figure 7A/B). As reflected by the rather similar zeta potential after incubation of the different ASP in protein-containing medium the protein corona seems to shield the charged ASP surface (Table 1).

### ASP efficiently develop a protein corona

**Figure 8:**
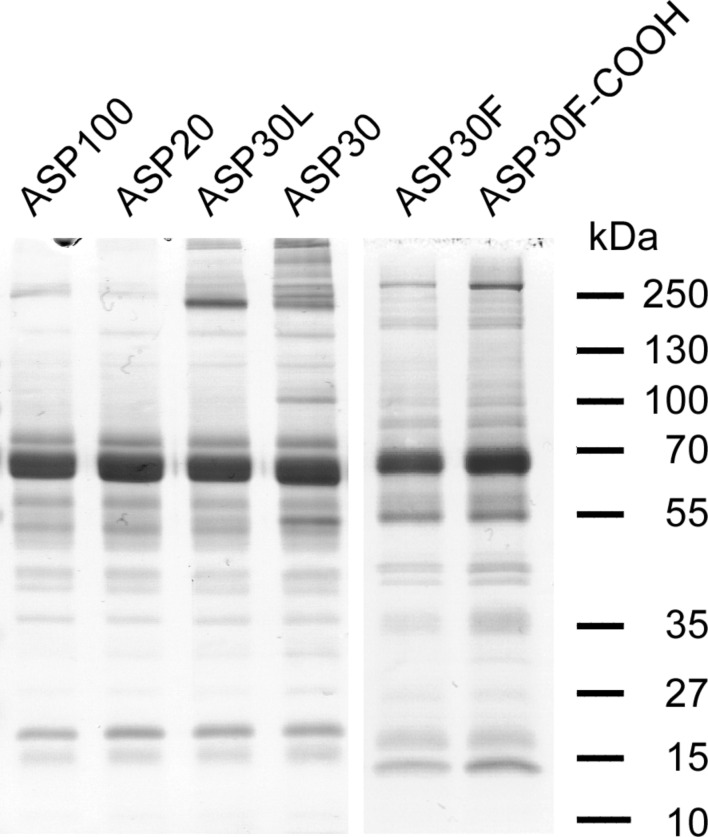
SDS-PAGE analysis to demonstrate the efficient formation of a protein corona around ASP. The indicated ASP suspensions were incubated with human plasma for 1 h. ASP–protein complexes were recovered by centrifugation, washed with buffer A, and the proteins were resolved by 1D SDS-PAGE. Corona proteins were visualized by Coomassie brilliant blue R-250 blue staining. MW is indicated.

As the presence of human plasma proteins attenuates ASP-induced toxicity, we examined whether the tested ASP are indeed able to adsorb proteins. Here, the particles were incubated with human blood plasma for one hour. Subsequently, the formed ASP–protein complexes were separated from excess plasma by centrifugation and washed to remove unbound residual plasma proteins. As shown in Figure 8, SDS-PAGE demonstrated that a stable and complex protein signature efficiently evolved on all particles tested. Besides the efficient adsorption of serum albumin, indicated by the prominent protein band of approximately 70 kDa, high molecular weight proteins were found to be enriched particularly on the ASP30 and ASP30L.

## Discussion

Besides ingestion, inhalation is one of the major entry routes for nanomaterials into the GI tract. Indeed, the majority of inhaled nanoobjects are transported out of the lung by the mucociliary clearance mechanism and swallowed afterwards [17–18]. With its large surface area of about 2000 m^2^, the GI tract is thus not only an interesting target for nanobiomedical applications but is almost constantly exposed to natural and engineered NP [2,18]. It is expected that in the future occupational and public exposure to silica and other NP will further increase because of their huge potential and rising applications in technology and nanobiomedicine. To date, there are several reports about the potential toxicity of silica NP in the GI tract, based on studies employing in vitro as well as in vivo murine models [36–38]. However, the mechanistic impact of the physico-chemical parameters of NP as well as of the protein corona is not yet resolved in detail [38–39]. To complement our understanding, we, here, investigated the potential cytotoxic effects of amorphous silica NP, differing in diameter but not in their negative surface charge. In contrast to other studies, we first performed a state-of-the-art particle characterization. The knowledge of the physico-chemical ‘bar code’ of the NP is an absolute prerequisite in order to link individual or multiple particle parameters, such as geometry, pore size or surface functionalization to the observed nanobiological effects.

The negative zeta potential, hydrodynamic diameter and colloidal stability of the ASP dispersions were obtained in water, salt-containing buffer, and cell culture medium. Hence, the tested concentrations of ions, carbohydrates and supplements, such as vitamins, did not significantly affect the ASP profiles. Albeit other (bio)molecules besides proteins are known or at least discussed to adsorb to NP [7,40], their impact on the physico-chemical behavior of NP has to be investigated individually. In contrast, the addition of serum or plasma proteins resulted in a significant increase in the average hydrodynamic diameter of the particles. Whereas the increase for the larger ASP100 (Ø = 100 nm) may be caused predominantly by the formation of a protein corona, the dramatic increase observed for the smaller ASP20 (Ø = 20 nm) indicates the additional agglomeration of single particles, which was reported also for other NP [41]. Likewise, both effects are occurring for the ASP30/ASP30L samples (Ø = 30 nm). Albeit both, ASP30 and ASP30L, show similar size and zeta-potential, we observed an increased binding of proteins with high molecular weight to the ASP30L, correlating with an increased hydrodynamic diameter. As these ASP were purchased from different vendors, our observation underlines the necessity to perform research with standardized materials [13,38]. Albeit the zeta potential of the four different ASP tested increased in the presence of serum proteins, the overall negative charge of the particles was maintained. Hence, the cell models used in our study were always facing negatively charged ASP, independent of the presence or absence of a protein corona. Thus, surface charge alone seems unlikely to be the only determinant of toxicity. Notably, we did not notice a significant difference in uptake by increasing the surface charge of ASP by carboxylation (ASP30F versus ASP30F-COOH). Hence, the protein corona seems to shield the ASP surface, as reflected by the rather similar zeta potential of the different protein-covered ASP. Whether the charge of pristine NP is indeed a general direct determinant of NP–cell interactions and uptake remains to be proven.

Notably, we observed here a time- and dose-dependent toxicity prudentially for the ASP30 or ASP30L in the absence of proteins. In contrast, exposure even to high doses (60 µg/mL) of either the smaller ASP20 or larger ASP100 particles under identical experimental conditions did not affect cell vitality, as convincingly shown by several independent experimental assays. Albeit previous studies also reported a toxicity dependent on the particle size of ASP in different cell models [19,42–43], it was postulated that the smaller particles always affected the exposed cells faster and at a lower dose. Thus, albeit these studies were conducted in the presence of low protein concentrations, it was suggested that the surface area and/or surface characteristics of the particles are unique parameters determining their toxicity [19–20,42–43]. Clearly, our results now demonstrate that the situation is more complex, and that the surface area alone is not a generally applicable predictive parameter for nanotoxicity. We found that in the absence of proteins slightly more ASPs were taken up by the Caco-2 cells compared to the incubation in the presence of proteins. The ‘physiological coating’ with proteins seems to influence the interaction of NP with the uptake machinery of the cell and thus, results in a reduced intracellular NP dose. However, whether reduced uptake is the (only) major determinant of the cytoprotective impact of the protein corona remains to be verified [44]. Clearly, the underlying mechanisms are not yet fully resolved. Of note, similar to the lung surfactant, the GI tract also contains additional biobarriers, such as mucous matrices and other biomolecules. Thus, future studies need to consider experimentally this layer of additional complexity to resolve the mechanisms and (patho)biological effects of silica nanoparticles in vitro and in vivo. Generally, the processes and molecular details involved in ASP toxicity are not yet resolved. Among other mechanisms, it was shown that ASP adsorb to cellular surfaces and can affect the structure, surface pressure, and integrity of membranes by physical and chemical interactions. Albeit often clear mechanistic insights were not fully provided, nanoparticle-induced toxicity was directly or indirectly linked to a variety of cellular (signaling) processes by numerous studies. Besides distinct cellular uptake mechanisms, the interaction with receptor molecules include also damaging effects on inner cell membranes, such as the mitochondria or ER. As a consequence, ASP–cell interactions may also trigger the production of reactive oxygen species (ROS), causing inflammatory responses and/or induce cell death [19–20,43,45]. Moreover, the direct binding of NP to proteins may additionally modulate downstream cellular signaling pathways, ultimately contributing to impaired cell vitality. In summary, close inspection of the literature reveals that the proposed mechanisms reported by different laboratories are not always consistent and sometimes even contradicting, underlining the need for further, ideally standardized studies. To this end, high-throughput testing is a key strategy to fill current gaps in knowledge and to systematically build nanomaterial structure–activity relationships (nanoSAR) [30]. By using adequate fluorescence microscopy imaging platforms, dual-color fluorescence cell vitality assay established here is, in principle, applicable to unbiasedly analyze the impact of other nanomaterials on multiple adherent cell models in an automated high-throughput fashion. NanoSAR will also aid to predict, which physico-chemical properties of the NP may potentially lead to nanopathology and thereby, reduce the use of animal models as the primary test platform [30].

We, here, clearly demonstrated by using independent experimental methods that the protein corona ameliorates ASP-induced toxicity in our cell model. Currently, the protein corona of nanomaterials in general is actively discussed as a major factor (co)determining their biological identity and hence, effects at the nano–bio interface [22]. In biological fluids, proteins bind to the surface of nanoparticles to form a biological coating around the nanoparticle, known as the protein corona. Over the time, this corona evolves and may modulate nanoparticle-induced processes such as opsonization which have direct consequences on the mode of interaction with cells, the efficacy of cellular NP uptake and thus, the organ targeting, biodistribution, and circulatio time of NP in vertebrates and non-vertebrates [22].

Our study indicates that the properties of the NP dictate the extent and speciﬁcity of protein binding proﬁles, which are complex, and in line with other studies may well consist of more than hundred different proteins [22,27–29]. Albeit we did not perform a detailed identification of the protein corona composition by comprehensive proteomic methods, previous studies demonstrated that the coronae indeed contain (patho)biologically relevant proteins [22,27–29]. Such proteins are not only involved in essential processes of the blood system and, thus, may act as opsonins or dysopsonins and modulate blood coagulation, but are also implicated in (cytoprotective) signal transduction pathways [22,28–29,46]. Facing the complexity of the plasma corona, one main challenge is clearly to determine to what extent the nanobiological effects observed are mediated directly by the biological activity of corona proteins, and to dissect the involved molecular mechanism. Additionally, the impact of the formation of a corona on transforming the primary properties of the formulated nanomaterials needs to be investigated. Notably, further studies need to consider also the impact of GI fluids or mucus on the biotransformation of ingested nanomaterials. Albeit these investigations will be challenging, the results of such studies are required to complete out understanding of the fate of nanomaterials in physiologically relevant environments.

In conclusion, based also on our study it is conceivable to speculate that depending on the type of nanomaterials the presence of proteins will have dual effects: For one, proteins may trigger the formation of a biologically active protein corona. Second, by lowering the effective particle dose by aggregation or by reducing local surface charge, proteins may change the physico-chemical properties of the NP. As both effects will ultimately be relevant for nano–bio responses, interdisciplinary efforts on a technologically advanced level are needed to better understand and predict nano(patho)biology.

## Experimental

### Characterization of amorphous silica nanoparticles (ASP)

The different aqueous dispersions of ASP were purchased from Nyacol Nano Technologies (ASP20, ASP30, ASP100), Sigma (Sigma-Aldrich, Taufkirchen, Germany) (ASP30L) or Kisker Products (ASP30F, ASP30F-COOH) and used as received. The ASP were characterized with respect to shape, size, and size distribution in the dry state as well as in solution. Transmission electron microscopy imaging was performed by using a Philips EM420 on carbon-coated copper grids as outlined in [47–48]. The size and zeta potential for the ASP were determined with a Malvern Zetasizer NanoZS as described in [29,49]. ASP were diluted with water, buffer A (103.5 mM NaCl, 5.3 mM KCl, 5.6 mM Na_2_HPO_4_, 1.4 mM KH_2_PO_4_, 23.8 mM NaHCO_3_, pH 7.4), DMEM with or without 10% FCS, and the measurements were conducted at 25 °C by using 0.6 mg/mL ASP.

### Cell culture

The colonic carcinoma cell line Caco-2 and the colorectal adenocarcinoma cell line HT-29 were obtained from American Type Culture Collection (ATCC HTB-37 and ATCC HTB-38, Rochville, USA) and grown in a 5% CO_2_ humidified atmosphere at 37 °C in DMEM medium (Life Technologies, Carlsbad, USA) supplemented with 10% FCS, 1% L-glutamine and 1% Pen/Strep (100 U/100 μg/mL) as described in [47]. 24 h prior to ASP exposure 2.0 × 10^4^ cells/well were seeded from a confluent culture flask into 96 well plates.

### Exposure of cells to ASP

The cells were exposed to different concentrations of ASP in either serum-free or serum-containing medium. To avoid the aggregation of the nanoparticles pre-dilutions of the ASP dispersion were made in MilliQ water (Millipore, Billerica, USA). Prior to ASP exposure, cells were washed with serum-free DMEM, and ASP dilutions were applied in serum-free DMEM. All dilutions were applied 1:10 in the respective medium to the cells. To investigate the effects of serum proteins, ASP working solutions were made and applied to the cells in serum-containing cell culture media. After an exposure time of 4 h, the cells were either analyzed or washed twice with DMEM and cultured for a further 4 h or 20 h time period.

### Human plasma

Human plasma was prepared as described [29]. Plasma aliquots were used only once to avoid protein degradation by multiple freeze-thawing cycles. After thawing, the plasma was centrifuged for 2 min at 12,000 rpm/4 °C to remove potential precipitates.

### ASP incubation with human plasma

ASP were incubated as described in [29,50]. Briefly, particle suspensions (60 µg/mL) were incubated with human plasma for 1 h at 4 °C (total volume 500 μL). The samples were centrifuged to pellet the particle–protein complexes (10 min at 12,000 rpm/4 °C). The pellets were resuspended in buffer A, transferred to a new vial, and centrifuged again to pellet the particle–protein complexes (10 min at 12,000 rpm/4 °C); this procedure was repeated three times. After the third washing step, the supernatant did not contain any detectable amount of protein. Proteins were eluted from the particles by adding SDS sample buffer (62.5 mM Tris-HCl pH 6.8; 2% w/v SDS, 10% glycerol, 50 mM DTT, 0.01% w/v bromophenol blue) to the pellet and incubation at 95 °C for 5 min.

### 1D SDS-PAGE

Discontinuous SDS-polyacrylamide gel electrophoresis (PAGE) was carried out according to standard procedures [51]. Proteins were visualized by staining with Coomassie brilliant blue R-250 as described [52]. All experiments were conducted at least twice to ensure reproducibility of the results.

### Statistical analysis

For experiments that state *P* values, a paired Student's *t*-test was performed as described in [47]. *P* values smaller than 0.05 were considered to be significant.

### Microscopy and imaging

After exposure to ASP observation of living cells, image analysis and presentation were performed as described in detail in [53].

### Measurement of cell viability

Cell viability was determined by using the electric sensing zone method (CASY^®^ TT Cell Counter; Schärfe SystemGmbH, Reutlingen, Germany) or by the mitochondria-dependent reduction of 3-(4,5-dimethylthiazol-2-yl)-2,5-diphenyltetrazolium bromide (MTT) assay as described in [54]. Briefly, following NP exposure, cells were incubated with MTT (400 μg/mL; 965 μM; Life Technologies, Carlsbad, USA) for 4 h. The MTT was removed, the cells were washed with PBS and solubilized in dimethyl sulfoxide (100 μL). The formazan was measured at 570 nm with a reference wavelength of 690 nm by using a plate reader (Thermo Fisher Scientific Inc., Berkshire, UK). Readings were background corrected with absorbance from maintenance media or NP in maintenance media without cells.

### Cellomics ArrayScan^®^ VTI-based high content screening (HCS)

Automated analysis of the cell viability assay was performed by using the Cellomics ArrayScan^®^ VTI Imaging Platform (Thermo Fisher Scientific Inc., Berkshire, UK). Cells were seeded with an electronic multichannel pipette (Eppendorf, Hamburg, Germany) into black-walled 96 well thin bottom Greiner µclear^®^ plates (Greiner, Frickenhausen, Germany) and incubated at 37 °C, 5% CO_2_ and 95% humidity. Cells were exposed to different ASP30 concentrations (0.6, 6, 60, 600 µg/mL). Cell viability was evaluated by our two-colour fluorescence cell viability assay using calcein-AM and ethidium homodimer-1 (Molecular Probes, Eugene, USA). Live (green) or dead (red) fluorescent cells were identified by fluorescence microscopy as described in [55]. Each experiment was performed in triplicate. PBS-treated cells served as negative and methanol-fixed cells as positive control (dead cells). Nuclei were stained by addition of Hoechst 33342 at a final concentration of 40 µM for 10 min. Images were acquired and analyzed on the Cellomics ArrayScan^®^ VTI Imaging Platform as described in [31]. Briefly, for every cell a binary image mask was created from the Hoechst 33342 staining signal to define the region of interest (ROI), resembling the nucleus. Intensity of calcein and EthD-1 signal were monitored within this mask. Scans were performed sequentially with settings to give subsaturating fluorescence intensity, and a minimum of 500 objects per well was recorded.

Automated analysis to quantify nanoparticles uptake was performed by using the Cellomics ArrayScan^®^ VTI Imaging Platform (Thermo Fisher Scientific Inc., Berkshire, UK) as described in [31]. Briefly, cells were seeded into black-walled 96 well thin-bottom µClear plates (Greiner) and further cultivated for 24 h. Cells were washed with PBS and either protein-free DMEM medium or DMEM containing 10% human plasma was added, and cells were exposed to 100 µg/mL fluorescent nanoparticles (ASPF30) for 60 min. Subsequently, cells were washed with PBS, fixed for 15 min with 4% paraformaldehyde and the cell nuclei were stained with Hoechst 33342. To quantify the amount of cell-associated nanoparticles, images were analysed by using Target Activation V4 assay [56]. For every cell, a binary image mask was created from the Hoechst 33342 staining signal to define the region of interest, marking the nucleus. In the second channel (red), this circular mask was dilated (four pixels) to cover the whole cell, and the intensity of the red fluorescence signal was quantified within this mask. Scans were performed with settings to give sub-saturating fluorescence intensity. A minimum of 1,000 cells per well was recorded. PBS-treated cells served as a negative control to correct for background fluorescence.
